# Cellular Senescence in Endometrium: A Pivotal Regulator in Physiological Remodeling and Pathological Disorders

**DOI:** 10.7150/ijbs.123036

**Published:** 2025-10-20

**Authors:** Zi-Yang Yan, Wen-Jie Zhou, Jiang-Feng Ye, Feng Xie, Chun-Xue Zhang, Ming-Qing Li

**Affiliations:** 1 Department of Reproductive Immunology, The International Peace Maternity and Child Health Hospital, School of Medicine, Shanghai Jiao Tong University, Shanghai 200030, China.; 2 Shanghai Key Laboratory of Embryo Original Diseases, Shanghai 200030, China.; 3 Reproductive Medical Center, Department of Obstetrics and Gynecology, Ruijin Hospital, Shanghai Jiao Tong University School of Medicine, Shanghai 200025, China.; 4 Institute for Molecular and Cell Biology, Agency for Science, Technology and Research, Singapore.; 5 Medical Center of Diagnosis and Treatment for Cervical Diseases, Obstetrics and Gynecology Hospital of Fudan University, 200433, Shanghai, China.; 6 Central Laboratory, The International Peace Maternity and Child Health Hospital, School of Medicine, Shanghai Jiao Tong University, Shanghai 201112, China.

**Keywords:** senescence, endometrium, decidualization, endometriosis, intrauterine adhesions, thin endometrium, implantation failure, spontaneous abortion

## Abstract

As a highly dynamic tissue, the endometrium undergoes complex remodeling during the menstrual cycle and pregnancy. Recent studies have revealed that cellular senescence plays a pivotal role in both physiological renewal (e.g., menstrual shedding, decidualization) and pathological disorders (e.g., endometriosis, intrauterine adhesions, thin endometrium) of the endometrium. Under physiological conditions, senescent cells contribute to tissue repair and embryo implantation through precise regulation. However, pathological accumulation of senescent cells drives chronic inflammation, fibrosis, and reproductive dysfunction. Here we aim to summarize the mechanism indicating endometrial senescence and elucidating their pleiotropic roles in both physiological homeostasis and pathological progression, while discussing emerging therapeutic strategies for clinical translation—including senolytics and SASP inhibitors.

## 1. Introduction

The endometrium is a multicellular mucosal tissue lining the uterine cavity, composed of epithelial cells, stromal cells, immune cells, and other cellular components. Functionally, the human endometrium comprises two distinct layers: the hormone-responsive functional layer that undergoes cyclical shedding, and the basalis layer responsible for physiological regeneration 1. Across the proliferative, secretory, and menstrual phases, estradiol drives epithelial and stromal proliferation, while progesterone induces decidualization of endometrial stromal cells (ESCs) in response to signals such as cAMP 2-4. Decidualization is strictly temporally regulated: during a brief proinflammatory phase, ESCs secrete chemokines and interleukins supports endometrial receptivity followed by a strictly time-locked 2-4-day implantation window 1,5-8, providing an appropriate niche for embryo implantation 9. Upon embryo implantation, this specialized tissue provides critical support for embryonic development while protecting maternal tissues from excessive trophoblast invasion 3. During this stage, local immunity shifts toward an anti-inflammatory, tolerogenic milieu 10-12. Missing the critical window significantly elevates the risk of adverse pregnancy outcomes 13. Thus, endometrium serves as the cornerstone of reproductive health while simultaneously representing a potential site for pathological development 14,15. Endometrial dysfunction can lead to various pathological conditions including endometriosis, implantation failure, and spontaneous abortion 16, significantly impacting both fertility and quality of life for numerous women of reproductive age. Physiologically, within these cyclical transitions, the tissue deploys a transient, rapidly cleared senescence program that facilitates decidualization, shedding, and repair; when clearance fails or pro-senescent cues persist, cellular senescence becomes persistent, promoting inflammation, fibrosis, and immune imbalance that link normal cycling to pathology.

By contrast, within the endometrium cellular senescence is not inherently detrimental. Cellular senescence represents an irreversible cell cycle arrest state that occurs in diverse physiological and pathological processes, including tissue remodeling, injury response, fibrosis, carcinogenesis, and maintenance of immune microenvironment homeostasis. Recent studies have demonstrated that the high turnover state of the endometrium is closely associated with dysregulation of cell cycle control, and cellular senescence may play a pivotal role in various endometrial-related pathologies. Nevertheless, current research is hampered by small sample sizes, reliance on in vitro systems, and limited in vivo or translational validation. Moreover, the senescence-associated secretory phenotype (SASP) mechanisms in endometrium remain incompletely defined, and standardized senescence markers in the endometrium are lacking, complicating cross-study comparisons. These gaps underscore the need for further investigation on endometrial senescence. Here, we review cellular senescence in endometrial diseases, highlighting the current understanding of senescence signatures and molecular mechanisms, and discuss the potential of emerging therapeutic strategies such as senolytics and SASP inhibitors for clinical translation.

## 2. Endometrial Senescence: Hallmarks and Endometrium-Specific Features

### 2.1 Hallmarks and Signaling of Cellular Senescence

In vitro cultured endometrial senescent cells (SNCs) typically exhibit characteristic morphological alterations, including cellular enlargement, flattened morphology, and cytoplasmic vacuolization^7^,^18^. In contrast, in vivo senescent cells often maintain their normal morphology as determined by the surrounding tissue architecture. At the molecular level, characteristic senescence-associated alterations serve as definitive biomarkers for identifying cellular senescence 19. Senescent cells characteristically exhibit elevated expression of senescence-associated β-galactosidase (SA-β-gal), which is widely utilized for senescence identification. Additionally, molecular components of senescence-associated signaling pathways - particularly the gene and protein expression of p16 (CDKN2A) and p21 (CDKN1A) are considered classical biomarkers of cellular senescence. SNCs maintain active secretory capacity, releasing a spectrum of bioactive molecules including interleukins, chemokines, and growth factors that collectively constitute SASP. This SASP generates inflammation-related signals resembling immune responses 20 and mediates paracrine interactions with the extracellular microenvironment.

Multiple studies have demonstrated that SASP plays a dual role: Acute senescence transiently generates SASP components that recruit immune cells for rapid clearance of SNCs, thereby facilitating tissue remodeling processes such as embryonic development and wound healing 19. However, excessive cellular senescence or impaired clearance leads to chronic senescence 21,22, where in persistent SASP signaling establishes a proinflammatory microenvironment, promotes tissue fibrosis, and potentially facilitates tumorigenesis 22,23.The SASP amplifies local inflammatory responses through secreted cytokines such as IL-1β, IL-6, and IL-8, which activate NF-κB and JAK/STAT signaling pathways. This process may contribute to the establishment of a chronic inflammatory microenvironment and facilitate senescence propagation to neighboring cells 21.

Through the secretion of chemokines (e.g. CCL2 and CXCL1), SASP recruits immune cells such as macrophages, T cells, and NK cells. These recruited cells subsequently release additional inflammatory factors and reactive oxygen species (ROS), thereby exacerbating both local and systemic inflammatory responses 24. SASP also mediates extracellular matrix (ECM) remodeling through the secretion of proteases and matrix metalloproteinases (MMPs), thereby disrupting tissue integrity and promoting cancer metastasis 23. Furthermore, SASP derived profibrotic factors, including transforming growth factor-β (TGF-β) and plasminogen activator inhibitor-1 (PAI-1), directly drive fibrotic progression 25,26.

### 2.2 Endometrium-specific features and physiological roles

**Figure 1:**
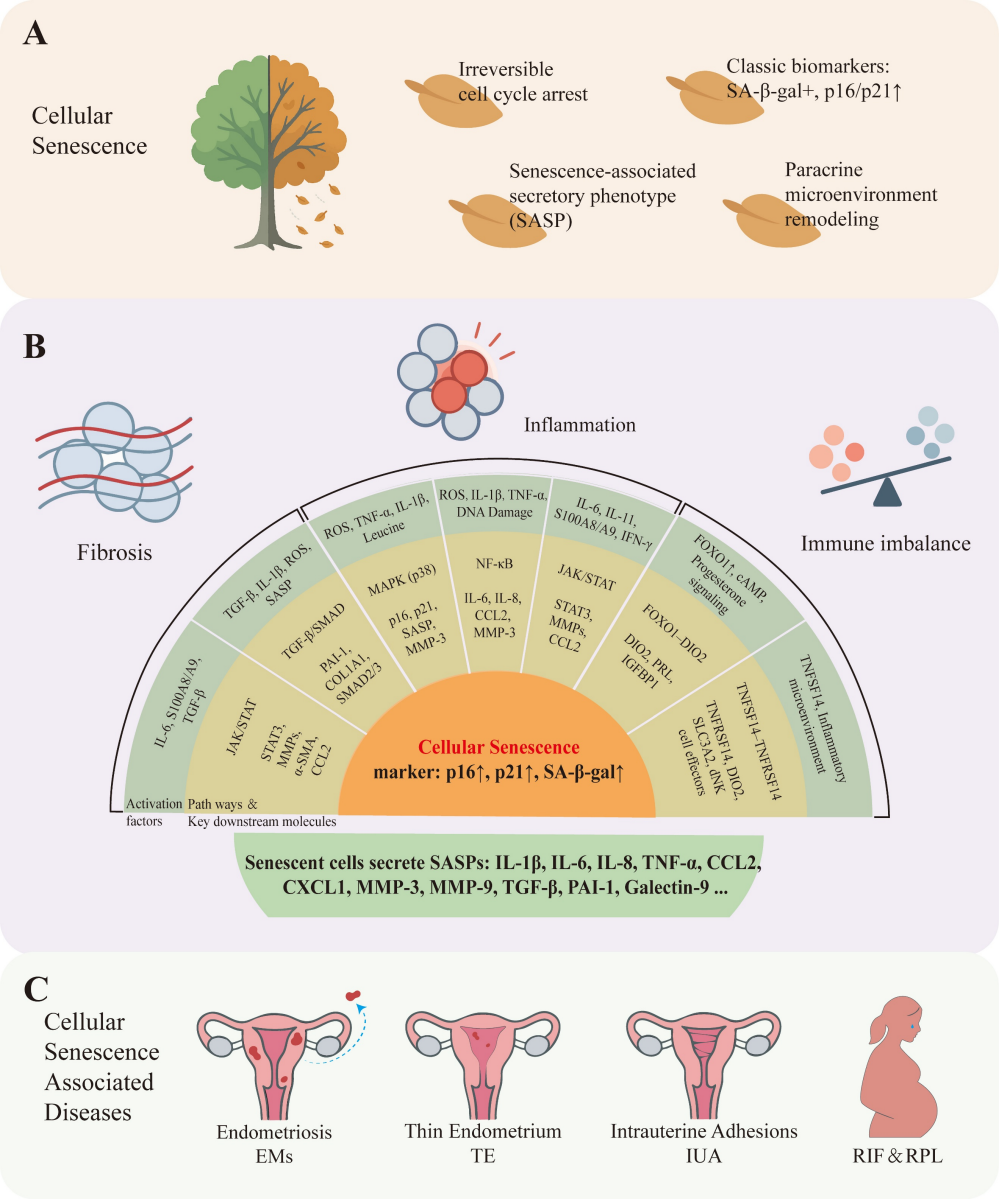
** Cellular senescence markers in endometrium and endometrium-related disorders**. A: According to existing research, cellular senescence has the following characteristics: Irreversible cell cycle arrest, Classic biomarkers such as SA-β-gal positive, p16/p21 upward, secrete a variety of cytokines collectively constitute the senescence-associated secretory phenotype (SASP), remodeling the cellular microenvironment through paracrine signaling. B: Upstream signaling pathways that drive cellular senescence in the endometrium can be grouped by functional role into three categories: 1 inflammation-related pathways, e.g., MAPK (mitogen-activated protein kinase), NF-κB (nuclear factor kappa-B), and JAK/STAT (Janus kinase / signal transducer and activator of transcription); 2 fibrosis-related pathways, e.g., TGF-β (transforming growth factor-β) and downstream SMAD signaling; and 3 immune-imbalance-related axes, e.g., the FOXO1-DIO2 axis and TNFSF14-TNFRSF14 signaling. Activation of these pathways promotes entry into a senescent state. Typical senescence phenotypes include increased expression of p16 INK4a (CDKN2A), p21 CIP1 (CDKN1A), and senescence-associated β-galactosidase (SA-β-gal), together with secretion of senescence-associated secretory phenotype (SASP) factors (for example IL-1β, IL-6, IL-8, CCL2) that mediate inflammation, extracellular matrix remodeling, and paracrine senescence. C: Numerous studies have shown that cellular senescence can cause many endometrial diseases such as endometriosis, thin endometrium, intrauterine adhesions, recurrent implantation failure and recurrent pregnancy loss.

The endometrium, as a highly dynamic tissue, undergoes approximately 400 cyclical changes during a woman's reproductive lifespan 14. Its periodic regeneration and shedding rely on precisely regulated mechanisms of cellular proliferation, differentiation, and clearance. Recent studies have revealed that controlled cellular senescence occurs during endometrial tissue turnover, serves dual roles: maintaining tissue homeostasis and contributing to functional disorders such as infertility, recurrent implantation failure (RIF), and recurrent pregnancy loss (RPL) 1 (Figure 1). The unique physiological context of the endometrium imparts specific characteristics to its senescent cells. For instance, endometrial senescence is tightly coupled to steroid hormone fluctuations, particularly the withdrawal of progesterone, which acts as a key trigger for senescence-associated inflammatory responses during menstruation 15,27. Furthermore, the senescence program in decidualizing stromal cells is orchestrated by the FOXO1-DIO2 axis and is crucial for establishing endometrial receptivity and facilitating embryo implantation 7,28. Following the initiation of decidualization, transcription factors such as FOXO1 induce the expression of decidualization marker genes, including prolactin (PRL) and insulin-like growth factor binding protein 1 (IGFBP1) 1,29. The immune microenvironment of the endometrium, rich in specialized uterine natural killer (uNK) cells, is particularly adept at surveilling and clearing senescent cells, a process critical for cyclical renewal and pregnancy maintenance 7,16. Therefore, positioned at the interface between physiological renewal and pathological remodeling, endometrial senescence serves as a key mechanistic nexus connecting cellular senescence with both physiological and pathological processes in endometrium.

## 3. Characteristics of Cellular Senescence in Physiological Endometrium

### 3.1 Menstrual Cycle Dynamics and Cellular Senescence

During the proliferative phase, rising estrogen levels drive endometrial proliferation, with mitotic activity being the most prominent feature of stromal and perivascular cells 27,30. Estrogen upregulates telomerase activity 31, thereby delaying cellular senescence. Upon entering the secretory phase, progesterone becomes the dominant regulatory hormone that drives endometrial differentiation to prepare for potential embryo implantation. The most prominent morphological change during this phase is the initiation of ESC decidualization. Following successful embryo implantation, cellular senescence within decidualized cells plays critical roles in modulating embryonic invasion depth and establishing local immune tolerance.

During the menstrual phase, endometrial cells progressively enter a senescent state accompanied by localized inflammatory responses and tissue remodeling, which collectively facilitate the shedding of the functional layer 27. Apoptosis plays a pivotal role in this phase by eliminating accumulated SNCs from the functional layer, thereby maintaining endometrial cellular homeostasis 32. Single-cell transcriptomic analyses have demonstrated that by day 7 post-luteinizing hormone surge (LH+7), corresponding to implantation window, ESCs exhibit significantly elevated senescence markers. This increase in senescence, coupled with moderate secretion of inflammatory factors, commonly prepare for the decidualization. By the premenstrual phase (LH+11), senescence signals intensify further and demonstrates a significant correlation with localized inflammation. 1,33. Progesterone withdrawal relieves the suppression of the NF-κB pathway 15, leading to upregulation of specific inflammatory factors by SNCs 2,14. These inflammatory mediators subsequently recruit macrophages and neutrophils, which in turn secrete MMPs (including MMP-9) to facilitate ECM degradation and subsequent shedding of the functional layer 34. Uterine natural killer cells (uNK) possess the capacity to recognize and eliminate senescent decidual cells, thereby fulfilling a critical immune surveillance function 7. This cyclical senescence process, coupled with subsequent inflammatory-repair mechanisms, forms an intrinsic regulatory circuit governing endometrial shedding and regeneration. This unique physiological program enables complete renewal of the functional layer, representing a distinct biological phenomenon compared to scar-forming repair processes characteristic of other tissues 35.

### 3.2 Decidualization and Cellular Senescence

During the mid-luteal phase, a series of programmed changes occur to facilitate embryo implantation and pregnancy maintenance, involving cellular differentiation, matrix remodeling, and immune regulation 7. Accumulating evidence indicates that programmed cellular senescence constitutes an integral component of normal decidualization 1. During decidualization, a subset of stromal cells exhibits classical senescence markers, including stabilized p53 expression, upregulated p16, and detectable SA-β-Gal activity. These cells are operationally defined as senescent-like decidual cells (snDCs) 1. Experimental evidence demonstrates that controlled cellular senescence is essential for successful decidualization: rapamycin-mediated inhibition of senescence signaling concomitantly suppresses expression of decidualization marker genes PRL and IGFBP1^18^,^29^. Acute senescence of decidual cell subpopulations is induced by the FOXO1-DIO2 signaling axis 7,28. Knockdown of FOXO1 suppresses DIO2 expression and attenuates decidual senescence in ESCs, whereas FOXO1 activation upregulates DIO2 expression and promotes decidual senescence 28.

Furthermore, the orderly regulation of cellular senescence significantly impacts embryo implantation capacity. SnDCs exhibit a classic acute senescence phenotype 7, and their secreted SASP factors enhance the initial inflammatory response during decidualization, thereby inducing secondary senescence 36. On one hand, SASP factors promote moderate decidual expansion, induce the expression of implantation-essential factors, and recruit immune cells, thereby enhancing endometrial receptivity 1,37; On the other hand, SNCs alter the mechanical and immune properties of decidual tissue, providing the structural and spatial conditions required for embryo invasion 7. SnDCs recruit and activate decidual natural killer cells (dNKs), which eliminate SnDCs via exocytosis of perforin and granzymes. This process facilitates embryo penetration through structurally loosened regions of the decidual barrier, enabling successful invasion and implantation 38,39. Excessive suppression of cellular stress and senescent subpopulations during early decidualization may lead to over-stabilization of decidual tissue, resulting in insufficient tissue remodeling dynamics and ultimately impairing normal embryo implantation 18. However, excessive accumulation of uncleared SNCs in decidual tissue leads to overproduction of SASP factors, which triggers disproportionately strong inflammatory and matrix degradation responses 18. This subsequently attenuates decidual cell responsiveness to progesterone and elevates the risk of pregnancy failure 36,40.

In summary, cellular senescence-like changes during decidualization constitute a critical process for pregnancy establishment, where precise regulation is essential for successful embryo implantation and pregnancy maintenance 36. Dysregulated senescence may lead to decidualization defects, resulting in pregnancy complications such as implantation failure and recurrent miscarriage 28,41. Furthermore, the functional crosstalk between snDCs and dNK cells plays a pivotal role in maintaining immune homeostasis during early pregnancy 16.

## 4. Endometrial-Related Diseases and Senescence Characteristics in Pathological and Therapeutical Conditions

**Figure 2:**
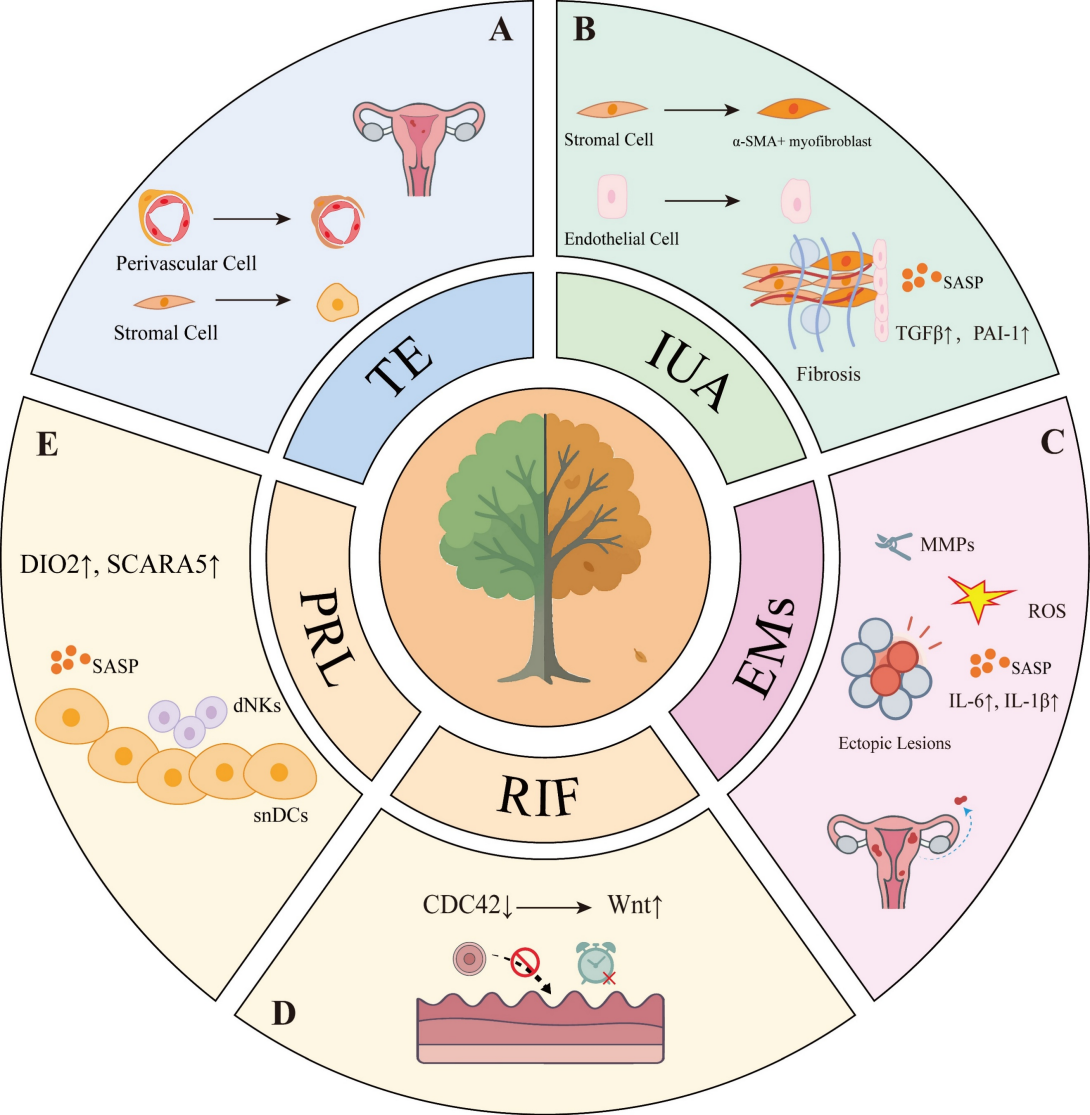
** Abnormal cellular senescence and distinct pathological features in endometrium-related disorders**. A: In TE, senescence of stromal cells and perivascular cells undergo aberrant senescence. B: In IUA, senescent endothelial cells release SASP factors, which collaborate with α-SMA-positive myofibroblasts to drive fibrosis. C: In EMs, senescent stromal cells sustain chronic inflammation through SASP components such as IL-6 and IL-1β, while ROS and MMPs further contribute to disease progression. D: In RIF, dysregulated temporal control of cellular senescence leads to impaired endometrial receptivity. E: In PRL, inadequate clearance of senescent decidual cells results in diminished endometrial receptivity.

Under physiological conditions, cellular senescence participates in the cyclic regeneration of the endometrium and the regulation of embryo implantation. However, excessive accumulation or impaired clearance of SNCs may contribute to various endometrial disorders (**Figure 2**). In thin endometrium, senescent basal stem cells lead to diminished regenerative capacity. In intrauterine adhesions (IUA), senescent stromal cells secrete pro-fibrotic factors (e.g., TGF-β, CTGF), driving excessive collagen deposition and uterine cavity obliteration. In endometriosis (EMs), ectopic lesions exhibit SNCs that sustain chronic inflammation and angiogenesis through SASP components (e.g. IL-6, MMP-3) (**Table 1**).

Although manifestations vary across endometrium-related disorders, a common feedback loop is widely recognized: the accumulation of senescent cells and persistent inflammation mutually reinforce each other. Senescent endometrial cells, via the SASP, secrete inflammatory cytokines such as IL‑1β, IL‑6, and TNF‑α that activate NF‑κB and p38 MAPK signaling, leading to stromal cell migration, neovascularization, and immune cell infiltration. Inflammation in turn provokes DNA damage, hyperactivation of mTOR, and oxidative stress, which further drives cellular senescence and SASP - establishing the senescence-inflammation feedback loop. Notably, agents such as quercetin, metformin, and rapamycin - despite targeting distinct pathways - have all been shown to exhibit activity against the senescence-inflammation axis and therapeutic efficacy in multiple endometrial disorders.

These agents represent disparate drug classes—quercetin as a senolytic flavonoid, metformin as a metabolic modulator, and rapamycin as an mTOR inhibitor—they converge on the same core regulatory axis of endometrial pathology: the senescence-inflammation cycle. Mechanistically, all three target key nodes of this loop, notably the mTOR/AKT pathway, NF-κB signaling, AMPK activation, and the p53 network 42. Through this unified axis, they share the capacity to clear or modulate senescent cell populations, blunt the pro-inflammatory SASP, and reprogram the local immune-metabolic microenvironment of the endometrium 43,44. In concert, quercetin eliminates senescent cells, metformin suppresses NF-κB/SASP signaling via AMPK activation, and rapamycin inhibits mTOR, together disrupting the self-perpetuating senescence-inflammation loop that drives endometrial tissue dysfunction 43,44. These shared actions explain the broad therapeutic potential of these agents across pathologically distinct endometrial disorders despite the divergent etiologies of these conditions 44. Indeed, their convergent efficacy underscores the centrality of the senescence-inflammation axis in driving diverse endometrial pathologies and supports this cycle as a unified therapeutic target in endometrial disease 44.

Collectively, these findings indicate that eliminating senescent cells, inhibiting the SASP, and modulating key signaling pathways can effectively disrupt this self-perpetuating “senescence-inflammation” cycle. The combined or optimized use of these interventions serves as a common, foundational therapeutic strategy across various endometrial disorders. In the following sections, we will summarize these strategies in the context of specific endometrial pathologies, and discuss their applications and benefits for each condition.

### 4.1 Endometriosis

EMs is a prevalent chronic inflammatory and hormone-dependent disorder characterized by the ectopic growth of endometrial tissue. Clinically, EMs primarily manifests as pelvic pain and infertility, affecting 5-10 % of reproductive-aged women worldwide and significantly impairing their quality of life 45,46.

Recent studies have revealed the presence of canonical cellular senescence features in endometriotic lesions 47. Specifically, deep infiltrating endometriosis (DIE) lesions exhibit significantly higher p16 expression compared to matched eutopic endometrium, along with elevated IL-1β levels, while further analyses reveal that peritoneal fluid and the peri-lesional microenvironment in these patients contain multiple proinflammatory cytokines—including IL-6, IL-8, and IL-1β—showing substantial overlap with the SASP secretory profile 48. Furthermore, studies on ESCs demonstrate that ESCs from EMs patients exhibit significantly higher SA-β-Gal activity even without external stimulation, whereas minimal SA-β-Gal staining is detected in healthy controls 49.

IL-1β, as an early-phase SASP factor, initiates downstream NF-κB signaling and accelerates ESC senescence via the JNK pathway 50. This cascade induces production of major SASP components such as IL-6 and IL-8, establishing a positive feedback loop that amplifies inflammation and propagates senescence: SASP factors further enhance local inflammatory responses while inducing secondary senescence in neighboring cells through paracrine mechanisms. Under physiological conditions, NK cells and macrophages effectively identify and eliminate SNCs 51. However, in EMs patients, the aberrant pelvic immune microenvironment enables SNCs to evade immune clearance and accumulate locally 52. Concurrently, these SNCs frequently exhibit an anti-apoptotic phenotype, resisting programmed cell death activation 53.

**Figure 3:**
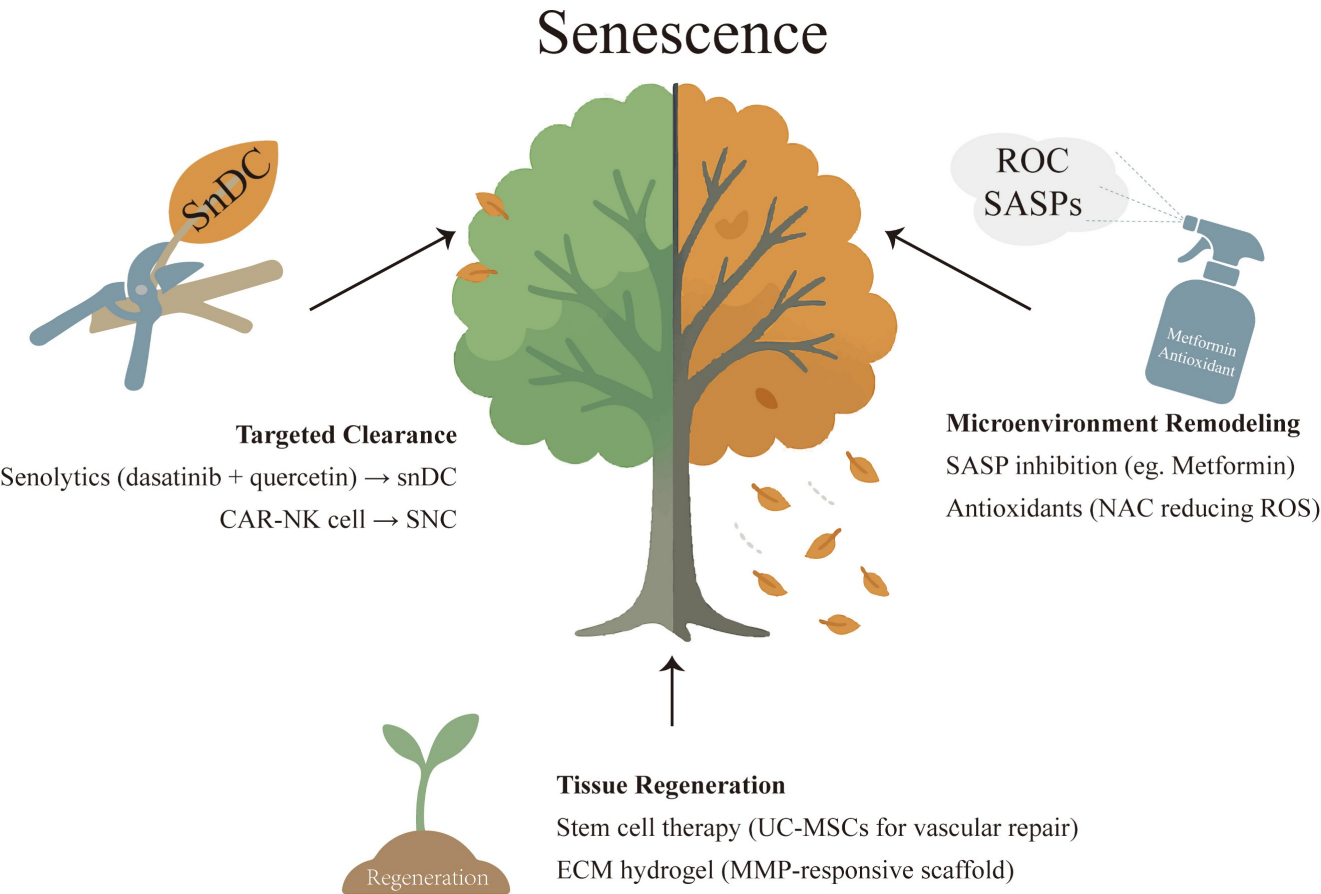
**Potential therapeutic strategies targeting cellular senescence in endometrial-related disorders**. Three main approaches: targeted clearance of senescent cells using senolytics (dasatinib and quercetin) to eliminate specific populations such as senescent dendritic cells; microenvironment remodeling via suppression of SASP and reduction of ROS with SASP inhibitors (metformin) and antioxidants (N-acetylcysteine), thereby attenuating chronic inflammation; and tissue regeneration by stem cell therapy (umbilical cord-derived mesenchymal stem cells) and ECM hydrogel (MMP-responsive scaffold).

Although the mechanistic role of cellular senescence in EMs pathogenesis remains incompletely understood, insights can be drawn from its established functions in tumor microenvironments. During tumor progression, senescent stromal cells establish an immunosuppressive microenvironment and drive tumorigenesis 54. These cells secrete proinflammatory SASP factors (e.g., IL-6, IL-1β) that sustain chronic inflammation 23, while simultaneously recruiting immunosuppressive cells (e.g., myeloid-derived suppressor cells, Tregs) to form an immunosuppressive microenvironment. This process not only inhibits NK cell function 55 but also enables tumor cells to evade immune surveillance. Concurrently, these senescent stromal cells release pro-angiogenic factors such as VEGF to support tumor vascularization 56. Furthermore, through the secretion of MMPs, they degrade the ECM, thereby facilitating tumor cell invasion and metastasis 57. As an invasive disorder, endometriotic lesions may concurrently harbor both proliferative cell populations and senescent yet metabolically active cells. The inflammatory mediators released by the latter could modulate the local immune milieu toward an immunosuppressive phenotype, thereby promoting cellular survival and contributing to lesion persistence and expansion. Accumulating evidence demonstrates that senolytic therapy can selectively eliminate pro-tumorigenic SNCs, with therapeutic benefits observed in diverse diseases including diabetes, Alzheimer's disease, and Parkinson's disease 58,59. As a classic senolytic cocktail, the combination of dasatinib and quercetin (D+Q) has shown efficacy in clearing SNCs and restoring tissue function across multiple disease models 60. In EMs, preliminary studies indicate that quercetin suppresses stromal cell proliferation while promoting normal differentiation in patient-derived cells, and reduces ectopic lesion size in animal models 61 (**Table 2**). Therefore, senolytic therapy holds potential to ameliorate the chronic inflammatory milieu in EMs by eliminating SNCs within ectopic lesions, which may partially restore healthy cellular function (see **Figure 3**). However, further experimental validation is required to assess its clinical translatability. Additionally, as SASP inhibitors, several anti-senescence drugs—including resveratrol, rapamycin, and metformin—have demonstrated therapeutic potential in inflammatory diseases. Future studies should explore their efficacy in alleviating endometriosis-associated pelvic inflammation 62-64.

ROS and iron overload also play significant roles in the progression of EMs. Compared to healthy women, EMs patients exhibit higher levels of iron in peritoneal fluid, may associated with retrograde menstruation and intra-lesional hemorrhage 65. These factors increase free iron levels, which through the Fenton reaction generate excessive ROS, further inducing oxidative stress damage 66. The accumulated ROS impose persistent oxidative stress on lesional cells, inducing senescence through multiple pathways including P38/MAPK and mTORC1 67. Furthermore, ROS trigger NF-κB pathway activation in peritoneal macrophages, exacerbating chronic inflammation and promoting angiogenesis within endometriotic lesions 68,69. ROS not only induce cellular senescence in both eutopic endometrium and ectopic lesions but may also disrupt the reproductive microenvironment. For instance, oxidative stress can cause spindle damage and telomere depletion in oocytes—mechanisms that are recognized as pivotal to oocyte aging and may contribute to endometriosis-associated infertility 70. Iron overload also drives cellular senescence and oocyte damage in ovarian endometriosis-associated infertility 71. Notably, ROS-scavenging interventions and iron overload mitigation have demonstrated potential to alleviate EMs symptoms in preclinical studies 62,71.

### 4.2 Thin Endometrium and Intrauterine Adhesions

Thin endometrium (TE) and IUA are common endometrial disorders associated with female infertility, both of which are closely linked to impaired endometrial regeneration. Emerging evidence suggests that cellular senescence may represent a shared pathological mechanism 72-74: On the one hand, senescent cell accumulation directly suppresses endometrial regeneration; On the other hand, SASP drives fibrotic progression.

#### 4.2.1 Thin Endometrium

TE is typically defined as a maximal endometrial thickness <7 mm during the proliferative phase, a condition associated with compromised endometrial receptivity, reduced implantation rates, and increased miscarriage risk 75. Single-cell RNA sequencing has revealed aberrant cellular senescence as a hallmark feature of TE 74. Specifically, the senescence of endometrial stem cells impairs endometrial proliferation, resulting in insufficient thickness to support embryo implantation 74. Furthermore, TE exhibits elevated p16 and p21 expression in epithelial cells, along with an increased proportion of senescent-associated elongated ciliated epithelial cells, indicating a pervasive senescent state within the endometrial epithelium 74,76. Endometrial mesenchymal stem cells (eMSCs), which reside in perivascular niches, normally possess high proliferative potential and multilineage differentiation capacity 77,78. However, in TE, perivascular cells demonstrate significant upregulation of senescence-related genes accompanied by impaired proliferation and differentiation, ultimately leading to reduced vascular density and compromised endometrial regeneration 30,74. Functional enrichment analysis further revealed that DIO2, a major key gene in the decidual senescence pathway, is significantly upregulated in thin endometrial stroma 30. Additionally, the abundance of uNK cells with senescent cell clearance capacity is markedly reduced in TE, leading to impaired senescent cell removal. This accumulation of SNCs perpetuates chronic senescence and excessive SASP production 79, which in turn promotes fibrotic progression. Recent findings further indicate that insufficient expansion or functional immaturity of cytolytic uNK cell subsets during the implantation window exacerbates the persistence of senescent stromal cells and local inflammation, thereby impairing endometrial receptivity and increasing the risk of implantation failure or pregnancy loss^80^.

Although occurring in distinct tissues, chronic wounds and TE share remarkably similar "senescence-regenerative dysfunction" mechanisms. In chronic wounds, excessive accumulation of senescent fibroblasts together with insufficient immune clearance establishes a persistent inflammatory milieu that suppresses angiogenesis and impedes repair 81,82. Senolytic therapy to eliminate SNCs has been shown to promote wound healing 81, implying that reducing the senescent burden or enhancing its immune clearance may be therapeutically relevant to TE. By contrast, current TE management has largely emphasized stem cell-based approaches to increase endometrial thickness and improve pregnancy outcomes, while the contributions of cellular senescence and immune microenvironmental change remain underexplored. Mechanistically, both conditions converge on a senescence-amplified loop; interrupting this loop by targeting senescent cells and recalibrating the immune microenvironment constitutes a testable therapeutic strategy for TE.

#### 4.2.2 Intrauterine Adhesions

IUA, also known as Asherman syndrome, refers to endometrial fibrosis following damage to the basal layer of the endometrium, resulting in partial or complete uterine cavity obliteration. This condition manifests clinically as menstrual abnormalities, infertility, and RPL. IUA is typically triggered by intrauterine procedures 83.

Studies have demonstrated that ESCs isolated from IUA patients exhibit significantly impaired colony-forming capacity, migratory/invasive potential, and angiogenic support compared to those from healthy women, displaying a premature senescence phenotype 84. Single-cell and transcriptomic analyses further reveal marked upregulation of senescence-associated genes and an increased proportion of SNCs in the proliferative-phase stromal compartment of IUA endometrium 72. Under stress conditions, ESCs undergo accelerated senescence and secrete abundant SASP factors, creating an immunosuppressive, pro-fibrotic inflammatory microenvironment 72,85. The SASP factor CCL2 is upregulated in IUA, recruiting immune cells including macrophages, a subset of which polarize into CD301+ pro-fibrotic phenotypes. These macrophages exacerbate endometrial fibrosis by activating the JAK/STAT signaling pathway 86. Concurrently, elevated levels of neutrophil-derived S100A8/A9 (a calcium-binding protein complex) promote ESC proliferation and differentiation into α-SMA-positive myofibroblasts via JAK2/STAT3 activation, driving excessive collagen and ECM deposition 87. Notably, galectin-9 (LGALS9), a lectin family protein highly expressed in senescent stromal cells of IUA, interacts with immune cell receptors to induce immunosuppressive effects, impairing the clearance of senescent and aberrant cells 72,88. Furthermore, stromal cell senescence may contribute to endometrial thinning, diminished ovarian hormone responsiveness, and reduced receptivity in patients 30,36. Collectively, the crosstalk between SNCs and inflammatory cells in IUA endometrium establishes a positive feedback loop, perpetuating a scarred microenvironment with impaired regenerative capacity.

Beyond ESCs, emerging evidence implicates endothelial cell (EC) senescence as a pivotal contributor to IUA pathogenesis. Endometrial cyclic regeneration critically depends on a robust microvascular network, which is disrupted in IUA due to basal layer damage and subsequent destruction of the decidua-associated capillary plexus, ultimately impairing tissue repair 89. At the molecular level, IUA patients exhibit significantly elevated expression of the senescence markers p16 and p21 in endometrial ECs compared to healthy controls 73. Single-cell sequencing and in vitro assays further confirm that microvascular ECs in IUA undergo pronounced senescence, characterized by diminished proliferative capacity and impaired angiogenic potential 73. These senescent ECs fail to adequately support endometrial revascularization, creating localized ischemia that activates stromal fibroblasts and promotes scar tissue deposition. Experimental studies using conditioned medium from senescent ECs to stimulate ESCs have demonstrated significantly elevated expression of fibrosis markers in the latter 73. Concurrently, ESCs from IUA patients overproduce PAI-1, which binds to the uPAR receptor on EC surfaces to induce endothelial senescence 73,90. Notably, TGF-β—already upregulated in early fibrosis stages—further amplifies PAI-1 production in ESCs via SMAD-dependent signaling, thereby establishing a self-reinforcing cycle 91,92. These findings position PAI-1 inhibition and other endothelial senescence-targeting strategies as promising therapeutic avenues for IUA prevention and treatment.

### 4.3 Recurrent Pregnancy Loss and Recurrent Implantation Failure

RPL and RIF are commonly associated with decidualization defects. Emerging evidence suggests that dysregulated temporal control of decidual senescence or impaired clearance of SNCs may compromise endometrial receptivity, ultimately contributing to these adverse reproductive outcomes 1,93,94. Mid-luteal phase endometrial biopsies from RPL patients reveal a "pro-senescent decidual response", characterized by upregulated expression of senescence-associated markers (e.g., DIO2, SCARA5) in the stroma. This excessive decidual senescence propensity correlates with elevated miscarriage risk 36. Decidual natural killer (dNK) cells—highly enriched during early pregnancy—normally surveil and eliminate aberrant cells. Studies demonstrate that dNK cells maintain decidual homeostasis by clearing SNCs, preventing their harmful accumulation 16. However, in RPL patients, insufficient dNK cell-mediated snDC clearance and impaired recognition of SNCs lead to excess snDC accumulation during decidualization, resulting in reduced endometrial receptivity 36,95. Endometrial tissues from RPL patients demonstrate elevated expression of DIO2 (a canonical snDC marker gene) alongside abnormal dNK cell accumulation, suggesting either expansion of decidual senescence phenotypes or a breakdown in senescence clearance homeostasis 96. In murine models, a high-leucine diet triggers extensive decidual cell senescence and subsequent embryonic loss 93. Branched-chain amino acids (e.g., leucine) accumulate in decidual stromal cells, activating the p38/MAPK pathway to drive cellular senescence 93,97. Conversely, TNFSF14-expressing dNK cells counteract leucine-induced senescence by engaging TNFRSF14 receptors on stromal cells 93. Notably, RPL patients exhibit enhanced decidual senescence, upregulated leucine transporter SLC3A2 while reduced TNFRSF14 expression. This pattern suggests coexisting leucine accumulation and defective anti-senescence regulation. Collectively, these findings demonstrate that dNK cells maintain decidual senescence homeostasis via the TNFSF14/TNFRSF14 axis.

In RIF patients, ESCs subjected to in vitro decidualization exhibit significantly lower levels of the classic SASP factor IL-6 and the snDC marker DIO2 compared to controls 98. Similarly, PAI-1—an established senescence marker 99—shows reduced expression in the endometrium of RIF patients 100. Notably, the proportion of p16-positive endometrial cells is markedly decreased in RIF women relative to those with successful pregnancies, suggesting that insufficient cellular senescence may paradoxically impair endometrial receptivity 101. Furthermore, primary ESCs isolated from patients with impaired endometrial receptivity during the proliferative phase demonstrate significant upregulation of SA-β-Gal activity and CDKN2A (the gene encoding p16), indicating premature ESC senescence 102. Studies have revealed reduced CDC42 (a GTPase) expression in RIF patient-derived ESCs 103. In vitro knockdown of CDC42 activates Wnt signaling and upregulates p21, thereby inducing cellular senescence and impairing decidualization—ultimately compromising uterine receptivity 103.

Clinical studies in both RIF and RPL patients have consistently documented ESC senescence signatures and disrupted endometrial immune microenvironments 102,104. Animal model investigations further confirm the critical role of senescence-regulating factors in decidual function 93,105. These collective findings highlight the therapeutic potential of targeting senescence pathways—such as snDC-clearing agents or TNFSF14-TNFRSF14 axis modulators—for improving endometrial receptivity and reducing RIF/RPL risk, representing novel intervention strategies.

## 5. Summary and Discussion

Emerging evidence indicates that, despite distinct phenotypic manifestations—chronic inflammation in EMs, regenerative impairment in TE, fibrosis in IUA, and receptivity defects in RIF or RPL—these disorders converge on a core pathogenic axis: aberrant accumulation of senescent cells coupled with dysregulated/failed immune surveillance. This axis integrates the senescence-associated secretory phenotype with persistent inflammation, dysregulated matrix remodeling, angiogenic imbalance, and regenerative failure, providing a coherent mechanistic framework that spans these conditions.

The therapeutic approach to endometrial disorders is undergoing a paradigm shift—from morphological restoration to mechanism-guided regulation. Traditional clinical studies have disproportionately focused on singular endpoints (e.g., excision of endometriotic lesions, lysis of intrauterine adhesions, or increasing endometrial thickness in TE), while overlooking the shared pathological axis of cellular senescence and immune microenvironment dysregulation. This mechanistic commonality unveils opportunities for cross-disease anti-senescence therapies, yet necessitates disease-specific adaptations. For instance, senolytics (e.g., dasatinib + quercetin) may effectively eliminate pathological senescent cells in endometriotic lesions, but require cautious application in RIF to avoid disrupting physiological decidualization, given the demonstrated role of controlled senescence in embryo implantation. Current anti-senescence strategies (e.g., senolytics, SASP inhibitors) have demonstrated therapeutic efficacy across multiple diseases. In EMs, the senolytic quercetin has shown promising results in animal models, significantly reducing lesion size and improving tissue remodeling. However, the precise mechanisms—particularly how combinations such as dasatinib plus quercetin selectively clear senescent ESCs while preserving physiological functions—remain incompletely elucidated. Senescence-directed CAR-T/NK approaches that improved myocardial fibrosis 86,106 suggest potential applicability to fibrosis-associated TE and IUA. In terms of immunomodulation, metformin shows cross-disease promise, with a large randomized controlled trial on “Metformin Targeting Aging” underway 107; in mice, metformin reduces the risk of spontaneous abortion by antagonizing decidual stromal cell senescence, indicating potential clinical value for senescence-driven endometrial disorders. A central caveat is the senescence paradox: physiological senescence is indispensable for cyclic endometrial remodeling and embryo implantation, whereas pathological senescence drives disease progression. For example, resveratrol's anti-inflammatory and anti-senescence actions may benefit pelvic endometriosis and uterine leiomyomas, yet simultaneously diminish decidual senescence and suppress PRL and IGFBP1 expression, thereby altering decidual programming 108. To enable precise clinical application, priorities include: 1 establishing endometrium-specific senescence biomarkers to identify treatment-responsive populations; 2 optimizing drug-delivery technologies to enrich therapeutic agents in diseased endometrial tissues; and 3 translating advances from anti-senescence drug development in other disciplines to launch cross-disciplinary clinical trials.

## Figures and Tables

**Table 1:** Characteristics and clinical correlations of cellular senescence in endometrial disorders

Disorder	Key Senescent Cell Types	Biomarkers	Pathological Consequences	Clinical Evidence
TE	Endometrial stem cells, Perivascular cells	p16↑, p21↑, DIO2↑	Impaired regeneration, Reduced vascular density	scRNA-seq shows senescence gene enrichment (74)
IUA	Stromal cells, Endothelial cells	PAI-1↑, TGF-β↑, Galectin-9↑	Fibrosis, Uterine cavity occlusion	EC senescence increases collagen deposition (73,90)
EMs	Ectopic lesion stromal cells	IL-6↑, MMP-3↑, ROS↑	Chronic inflammation, Angiogenesis	Higher p16 in lesions than eutopic endometrium (48)
RIF	Decidual stromal cells	IL-6↓, DIO2↓, CDC42↓	Decidualization defects, Impaired receptivity	Reduced p16+ cell proportion (101)
PRL	snDCs, Dysfunctional dNKs	DIO2↑, TNFSF14↓, SLC3A2↑	Excessive inflammation, Embryo rejection	Leucine accumulation in decidua (93,96)

**Table 2:** Therapeutic effects of anti-senescence agents in murine models of endometrium-related disorders

Drug/Therapy	Type	Anti-cellular senescence Mechanism	Indications as Anti-cellular Senescence Agent	Application in Endometrial Diseases	Ref.
Dasatinib & Quercetin	Senolytics	Inhibitor of BCL-2 and inhibitor of PI3Ks and serpins	Diabetic kidney disease, Idiopathic pulmonary	EMs, PRL	(44,60,61,109-113)
Metformin	SASP inhibition	Activates AMPK / inhibits NF‑κB / mTOR	T2DM, age-related disorders, inflammation	PRL, RIF, EMs	(41,64,93,109,114-116)
Rapamycin	SASP inhibition	Inhibits mTORC1 / reduces SASP	Age-related pathologies	EMs, RIF, RPL	(41,117-121)
Resveratrol	SASP inhibition	Activates SIRT1 / AMPK → NF‑κB suppression	NA	EMs, RIF	(108,122-124)
CAR-NK/T cell therapies	Senolytics	Immune-mediated clearance of senescent cells	Fibrosis, age-related disorders	NA	(125,126)

NA = no direct evidence in endometrial diseases; extrapolated from other indications
